# The socio-cultural factors behind the Saudi attitude toward COVID-19 vaccination: A survey-based study

**DOI:** 10.3389/fpubh.2022.1026252

**Published:** 2023-01-09

**Authors:** Muna Al-Ghuraibi, Ibrahim M. Dighriri, Mahmoud Essam Elrggal, Najla A. Obaid

**Affiliations:** ^1^Department of Social Studies, College of Arts, King Saud University, Riyadh, Saudi Arabia; ^2^Department of Pharmacy, King Abdulaziz Specialist Hospital, Taif, Saudi Arabia; ^3^College of Pharmacy, Umm Al-Qura University, Mecca, Saudi Arabia

**Keywords:** vaccination hesitancy, COVID-19 vaccine, accept vaccine, Saudi population, socio-cultural factors

## Abstract

**Introduction:**

Vaccine distrust and rejection are thought to contribute to disease outbreaks and increased mortality. The present study aimed to analyze the socio-cultural characteristics and attitudes of the Saudi population toward vaccines, using a cross-sectional survey-based approach.

**Methods:**

An online questionnaire was used, following the snowball method. A total of 444 people responded, of whom 333 (75%) were female, and 111 (25%) were male.

**Results:**

The demographic characteristics associated with vaccine confidence were gender, job type, medical problems, and knowledge of coronavirus disease 2019 (COVID-19) symptoms. The hesitancy was highest (31.17%) among individuals aged 21–30 years old, and in more males (27.03%) expressed hesitancy than females (25.23%). However, if we focused on the percentage of the refusal to receive the vaccine, more females (15.23%) refused the vaccine than males (4.5%). More than one-third of the vaccine-hesitant respondents had limited knowledge of COVID-19 symptoms. Personal characteristics associated with vaccine confidence were described as the following: do not fully trust vaccines produced in a short time (42.1%), fear of the future results of the vaccine (30.4%), reluctance to allow a foreign material to enter the body (17.6%), no interaction with others, so no need for the vaccine (11.5%), low interaction with people (67.8%), and reluctance to make decisions (11.3%). The primary social motivation for getting the vaccine was to get back to normal life (67.6%).

**Discussion:**

The results of the present study revealed that more than half of the respondents in Saudi Arabia were confident about the vaccine (61.7%), while only 25.7% were hesitant and 12.6% were unconvinced. Based on these results, in the early period of COVID-19 vaccine administration in the country (early 2021), before any governmental allowance and political intervention, we found that the socio-demographic and socio-cultural characteristics of the population were significant factors contributing to vaccination acceptance. Therefore, policymakers should support long-term safety studies of the vaccine, conduct educational programs giving high-priority to the populations' health, and tailor vaccination hesitancy reduction techniques to local communities.

## 1. Introduction

According to the World Health Organization (WHO) Strategic Advisory Group of Experts (SAGE), vaccine hesitancy is defined as “the refusal to vaccinate or the delay in accepting to vaccinate when a certain vaccine is available.” Another definition of hesitancy is the expression of concern or doubt regarding the value or safety of vaccination (1). Vaccines are intended to produce and enhance immunity in the human body against certain infectious diseases, while benefiting a group of people or an entire population. Such vaccines are offered at a specific time, aiming to decrease or eliminate the risk of infection (2). Lack of confidence in vaccination is a significant issue worldwide, and a growing lack of trust in vaccines, as well as a refusal to take the vaccine even when it is available, contributes to prolonged disease outbreaks and increased mortality (3). The broad range of information available drives attitudes for or against vaccination. People develop opinions on vaccinations from information gained through social media and online sources, and news reports, blogs, and other social media reviews have revealed an increasing number of Americans have rejected the coronavirus disease 2019 (COVID-19) vaccination (4). As a result, healthcare professionals face increased challenges in building trust in vaccinations. Developing vaccine confidence in the population means reassuring those who are hesitant and mitigating their concerns. During the approval process for COVID-19 vaccines, health officials monitored their safety and recorded any side effects that occurred (5).

With the onset of the COVID-19 pandemic in February 2020, and its subsequent worldwide spread, vaccine development was accelerated, bringing a viable vaccine to the market as soon as possible. Vaccine development and preclinical studies typically take 10–15 years; however, the COVID-19 pandemic presented an atypical situation. In October 2020, 92 vaccines were in preclinical phases, of which 43 were in phases I and II, 11 were in phase III, and 5 were approved. By the end of 2020, two vaccines had been approved for use in the United States, and vaccines were administered to frontline health professionals; in Europe, one vaccine had been approved, and the United Kingdom had begun to administer it to their population (6, 7); and in Saudi Arabia, one vaccine had been approved, and by the beginning of 2021, the Ministry of Health began a vaccination campaign in three major cities. According to the Ministry of Health, the campaign commenced in three phases in 2020. The first phase was to vaccinate frontline health professionals as well as those individuals in the most vulnerable categories, such as people with at least two chronic diseases, the elderly (those aged > 65 years), obese individuals with a body mass index (BMI) > 40%, and immunocompromised individuals. The second phase included individuals > 50 years old, obese individuals with a BMI of 40–30%, and individuals with one or more chronic diseases. The third phase was for any other individuals wanting a vaccine (8).

Many factors influence attitudes toward vaccination, some of which may be related to a lack of awareness and misinformation from different online and social media sources. Reasons for mistrust, however, appear more commonly in the literature than misinformation (1). Trusting the institutions that provide these vaccines, and honoring the values of the population the vaccine is aimed at is associated with vaccination confidence (9). The uptake of vaccines, including those developed during the pandemic, is a social endeavor involving human factors. Schoch-Spana et al. (4) believe that it is necessary to invest in research and investigate human factors, as well as social and behavioral findings, to ensure the success of any COVID-19 vaccination campaign (4). Cultural background plays a significant role in forming public opinions about vaccine risks and benefits (4), and little is known about how vaccine attitudes in Saudi Arabia are linked to those of other populations. Saudi studies are limited in their extent and assessment, and none have directly evaluated the attitudes of Saudi populations toward vaccines. One cross-sectional study, however, investigated the attitude toward COVID-19 vaccine acceptance among the Jordanian public (10), which was low (37%), but also found that participants who received the seasonal influenza vaccine were more likely to accept the COVID-19 vaccine. Participants indicated that healthcare providers were their most trusted sources of information on the COVID-19 vaccine (10).

Classifying the spectrum of attitudes toward vaccination presents a challenge. WHO classifies vaccination attitudes into three categories: (a) full acceptance; (b) full refusal; and (c) vaccine hesitancy (9). The vaccine hesitancy category lies between full acceptance and full refusal of vaccines, and as an “in the middle” category, those that fall here have some doubts about while accepting some vaccines, making it a heterogeneous category. The attitude toward the COVID-19 vaccine in Saudi Arabia requires a more robust investigation to identify specific community concerns. Qualitative research, therefore, may provide insight into the “how” and “why” of vaccine behavior, and may also capture public opinion (4). Sociocultural context has been found to play a significant role in attitudes toward vaccinations and the decision to vaccinate in many populations worldwide (9). Vaccination attitudes may be influenced by shared beliefs about the causes of diseases, as well as attitudes about modern medicine and local healthcare (11). It is crucial to explore any hesitancy toward COVID-19 vaccines and its predictors, as well as attitudes toward COVID-19 vaccines in general, among the Saudi population. The results of the present study can potentially assist policymakers in undertaking proactive campaigns and well-designed strategies to encourage vaccine acceptance and use by highlighting the importance of vaccination in the community.

## 2. Materials and methods

The present study explored various aspects associated with the COVID-19 vaccine in Saudi Arabia, and the significance of culture in the control of and fight against the ongoing COVID-19 pandemic. This research study is based on a descriptive cross-sectional study conducted on Saudi populations from different cities using the Hofstede model, which is suitable for explaining the sociocultural context behind individuals' decisions (12). This model includes six dimensions of national culture, including: power distance, uncertainty avoidance, individualism/collectivism, gender, long/short-term orientation, and indulgence/restraint. The primary feature of the present study was the diversity in participant selection.

A one-month period was planned to collect data after obtaining ethical approval (number: HAPO-02-K-012-2021-03-595). General public living in Saudi Arabia were recruited for the survey using the survey link generated using Google Forms application. The survey link was initially forwarded to general public by posting the link on community groups. Moreover, the survey was conducted for a period of 4 weeks from 24 March to 22 April 2021. As an approach to reach maximum samples in a short time, snowball sampling technique was adopted (13), in which a request is made while forwarding the survey link, whereby participants were requested to forward the message to their colleagues and friends. Accordingly, the survey link was targeting 600 people. As a result of using snowball sampling technique, we adapted Krejcie and Morgan module to calculate the representing sample size needed (14). With a margin of error of 5.0%, a confidence of 95% for a population size of 250,000 and over, the sample size is 384. Therefore, the final sample of 444 was achieved, indicating a response rate of 74%. Individuals of different ages, genders, social classes, education levels, job situations, and health conditions were considered in the sampling structure. The questionnaire consisted of the following three sections: the first section contained 11 questions regarding the sociodemographic characteristics of the participants; the second section included 18 questions related to the influence of personal characteristics on the decision to take the vaccine; and the third section included 15 questions that covered the social and cultural motivations behind individuals' decisions to accept, hesitate to accept, or refuse the vaccine. We used mean and standard deviation for continuous data and frequency/percentage for categorical data. Univariate analysis was used. Including Chi-square test was used when needed to test our hypothesis.

We analyzed the data using IBM SPSS Statistics for Windows, version 26 (IBM Corp., Armonk, N.Y., USA) to understand the sociocultural factors related to the Saudi culture and assess how to equip the people of Saudi Arabia to deal with the pandemic in a better way.

## 3. Results

### 3.1. Demographic characteristics of the participants

Survey respondents represented a random convenience sample of the Saudi population. There were 444 participants in the present study, of whom the overwhelming majority (333; 75%) were female, and only 111 (25%) were male, 30.2% were aged between 41 and 50 years old; 315 (70.9%) were married, 93 (20.9%) were single, and 26 (5.9%) were divorced. More than half of the respondents (56.5%) were bachelor's degree holders, and 20.9% were postgraduates. According to the results, more than half of the respondents were bachelor's degree holders, which represents the normal distribution of educational status in Saudi Arabia (15). Furthermore, 252 (56.8%) had permanent jobs, while 162 (36.5%) did not. A total of 228 (51.4%) did not have medical insurance, while 216 (48.6%) did. More than half of the participants (65.8%) had no medical problems, although 30.6% did. About half of the participants (231; 52%) were taking supplements, while 213 (48%) were not (Table 1).

**Table 1:** Demographic characteristics of the study participants (*n* = 444).

**Variable**		***n***	**Percentage**
Age (years)	< 20	12	2.7%
	21–30	77	17.3%
	31–40	89	20.0%
	41–50	134	30.2%
	51–60	108	24.3%
	>60	24	5.4%
Gender	Male	111	25.0%
	Female	333	75.0%
Social status	Single	93	20.9%
	Married	315	70.9%
	Divorced	26	5.9%
	Widowed	10	2.3%
Education	High school or less	47	10.6%
	Diploma	53	11.9%
	Bachelor's degree	251	56.5%
	Postgraduate studies	93	20.9%
Permanent job	No	162	36.5%
	Yes	252	56.8%
	Other (student or retired)	30	6.8%
Job type	Non-medical job	386	86.9%
	Medical job	58	13.1%
Medical insurance	No	228	51.4%
	Yes	216	48.6%
Medical problems	No	292	65.8%
	Yes	136	30.6%
	I don't know	16	3.6%
Take supplements	No	213	48.0%
	Yes	231	52.0%

### 3.2. The influence of personal characteristics on the participant's decision towards the vaccines

As shown in Table 2, 328 (73.9%) of the participants self-reported as having good knowledge of COVID-19 symptoms, although 108 (24.3%) had limited knowledge. Of the respondents, 12.4% had recovered from COVID-19, while 84% did not get it, and 3.6% were not sure. Moreover, of the 355 (80.0%) participants who had recovered from COVID-19, 61.7% were vaccine confident, 25.7% were hesitant, and 12.6% were not convinced. A total of 315 respondents did not have feelings against the vaccine (70.9%). Only 21.4% trusted vaccines that were produced in a short time; 30.4% were concerned about the vaccine's long-term effects; 72.7% did not agree that vaccines are conspiracies against humans; and 48.2% did not agree with the statement “I don't want foreign material to enter my body.” Most participants (80.9%) do not object to fate and destiny; however, 71.2% disagreed with the statement “I don't interact with others, so I don't need the vaccine;” 67.8% agreed that they do not mix with people a lot, so there is no need for a vaccine.

**Table 2:** Influence of personal characteristics on the decision to take the vaccine.

**Variable**		**Frequency**	**Percentage %**
Knowledge about COVID-19 symptoms	No knowledge	8	1.8%
	Limited knowledge	108	24.3%
	Good knowledge	328	73.9%
Previously infected by COVID-19	No	373	84.0%
	Yes	55	12.4%
	Maybe	16	3.6%
Anyone close infected by COVID-19	No	89	20.0%
	Yes	355	80.0%
Lost anyone close due to COVID-19	No	367	82.7%
	Yes	77	17.3%
Vaccine confident	No	56	12.6%
	Yes	274	61.7%
	To some extent	114	25.7%
I am against vaccines	Not agree	315	70.9%
	Somewhat agree	99	22.3%
	Agree	30	6.8%
I trust vaccines that are produced in a short time	Not agree	162	36.5%
	Somewhat agree	187	42.1%
	Agree	95	21.4%
I fear the future results of the vaccines	Not agree	137	30.9
	Somewhat agree	172	38.7
	Agree	135	30.4
I think it's a conspiracy against humans	Not agree	323	72.7%
	Somewhat agree	93	20.9%
	Agree	28	6.3%
I don't want a foreign material to enter my body	Not agree	214	48.2%
	Somewhat agree	152	34.2%
	Agree	78	17.6%
I do not object to fate and destiny	Not agree	30	6.8%
	Somewhat agree	55	12.4%
	Agree	359	80.9%
I don't interact with others, so I don't need the vaccine	Not agree	316	71.2%
	Somewhat agree	77	17.3%
	Agree	51	11.5%
I don't mix with people a lot, so there is no need for a vaccine	Not agree	18	4.1%
	Somehow agree	125	28.2%
	Agree	301	67.8%
Feel pessimistic about the future even after the epidemic is over	Not agree	364	82.0%
	Somehow agree	65	14.6%
	Agree	15	3.4%
Reluctance to make decisions is one of my traits	Not agree	284	64.0%
	Somehow agree	110	24.8%
	Agree	50	11.3%
In my opinion, every problem has a solution	Not agree	7	1.6%
	Somehow agree	79	17.8%
	Agree	358	80.6%
I always feel anxious, even for the most trivial reason	Not agree	239	53.8%
	Somewhat agree	145	32.7%
	Agree	60	13.5%
I would prefer that others take responsibility for me in making decisions	Not agree	335	75.5%
	Somewhat agree	87	19.6%
	Agree	22	5.0%
I felt depressed during the pandemic	Not agree	248	55.9%
	Somewhat agree	160	36.0%
	Agree	36	8.1%
I changed my mind more than once about the decision to take the vaccine	Not agree	284	64.0%
	Somewhat agree	106	23.9%
	Agree	54	12.2%
I am concerned about the side effects of the vaccine, which I heard about from a friend or relative	Not agree	263	59.2%
	Somewhat agree	181	40.8%

However, 82.0% of the participants did not agree that people felt pessimistic about the future, even after the epidemic was over; 64.0% did not agree that reluctance to make decisions was one of their traits; 80.6% agreed that every problem has a solution; 53.8% did not always feel anxious, even for the most trivial reasons; 75.5% said they did not agree that they would prefer that others take responsibility for their decisions for them; 55.9% did not agree that they felt depressed during the pandemic; 64.0% did not agree that they changed their minds more than once about the decision to take the vaccine; 59.2% did not agree that they were concerned about the side effects of the vaccine, which they heard about from a friend or relative (Table 2).

**Figure 1:**
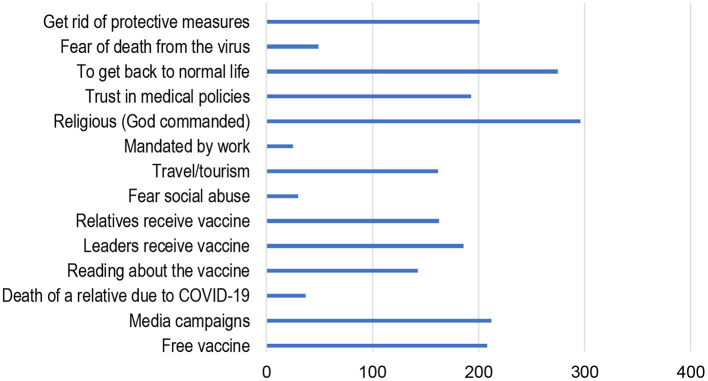
Social and cultural motivations behind individuals' decisions to take the vaccine.

The leading reasons that respondents took the vaccine were: religious/God commanded, 72.7%; return to normal life, 67.6%; media campaigns, 52.1%; and free vaccine, 51.1%. Moreover, participants said yes or no to taking the vaccine because of the following, respectively: leaders received the vaccine (45.7 vs. 54.3%); relative's death due to COVID-19 (9.1 vs. 90.9%); reading about the vaccine (35.1 vs. 64.9%); and relative's received the vaccine (40.0 vs. 60.0%). Other factors included: fear of social abuse (7.4 vs. 92.6%), travel and tourism (39.8 vs. 60.2%); obligated by work (6.1 vs. 93.9%); trust in medical policies (47.4 vs. 52.6%); fear death from virus (12.0 vs. 88.0%) and getting rid of protective measures (49.4 vs. 50.6%; Figure 1).

### 3.3. Demographic characteristics and the relationship to vaccine-confident among different socio-demographic characteristics

Other demographics did not have a statistically significant effect on vaccine confidence (*p* > 0.05), including age (*p* = 0.165), social life (*p* = 0.442), education (*p* = 0.153), permanent job (*p* = 0.428), medical insurance (*p* = 0.424), close to someone who developed COVID-19 (*p* = 0.346), and lost someone close due to COVID-19 (*p* = 0.135). The highest percentage of hesitancy was observed among those aged 21–30 years, and was higher in males (27.03%) than females (25.23%). The highest percentage of hesitancy (40.00%) related to social life was in the widowed population. Moreover, 27.16% of those who were vaccine hesitant did not have a permanent job, 27.63% did not have medical insurance, 37.96% had limited knowledge about COVID-19 symptoms, and 26.70% had not lost anyone close due to COVID-19. Most respondents who accepted vaccines demonstrated the following characteristics: aged between 41 and 51 years (66.42%); married (62.86%); worked in the medical field (72.41%); had medical problems (75.74%); had received a diploma (64.15%); had medical insurance (64.81%); had good knowledge about COVID-19 symptoms (66.16%); and had lost a close relative due to COVID-19 (71.43%). A large portion (76; 68.47%) of males were persuaded by vaccines, compared to five (4.50%) who were not. A total of 198 (59.46%) females were vaccine confident, compared with 51 (15.32%) who were not (Table 3). When comparing different professions regarding vaccine hesitancy; Non-medical profession were more hesitant than Medical profession with (OR = 8.2404, *P* = 0.039).

**Table 3:** The frequency distribution and chi-squared analysis of demographic characteristics and vaccine-confident among different socio-demographic characteristics of the study population.

**Variable**		**Vaccine confidence**			**P-value**
		**No** **(refusal)**	**Yes** **(acceptance)**	**To some** **extend** **(hesitant)**	
Age category	< 20	4 (33.33%)	7 (58.33%)	1 (8.33%)	0.165
	21–30	9 (11.68%)	44 (57.14%)	24 (31.17%)	
	31–40	14 (15.73%)	51 (57.30%)	24 (26.97%)	
	41–50	18 (13.43%)	89 (66.42%)	27 (20.15%)	
	51–60	10 (9.26%)	65 (60.19%)	33 (30.56%)	
	>60	1 (4.17%)	18 (75%)	5 (20.83%)	
Gender^*^	Male	5 (4.50%)	76 (68.47%)	30 (27.03%)	0.012
	Female	51 (15.32%)	198 (59.46%)	84 (25.23%)	
Social life	Single	12 (12.90%)	58 (62.37%)	23 (24.73%)	0.442
	Married	38 (12.06%)	198 (62.86%)	79 (25.08%)	
	Divorced	6 (23.08%)	12 (46.15%)	8 (30.77%)	
	Widow	0 (0.00%)	6 (60.00%)	4 (40.00%)	
Education	High school and less	7 (14.89%)	27 (57.45%)	13 (27.66%)	0.153
	Diploma	5 (9.43%)	34 (64.15%)	14 (26.42%)	
	Bachelor's degree	37 (14.74%)	160 (63.75%)	54 (21.51%)	
	Postgraduate studies	7 (7.53%)	53 (56.99%)	33 (35.48%)	
Permanent job	No	26 (16.05%)	92 (56.79%)	44 (27.16%)	0.428
	Yes	26 (10.32%)	163 (64.68%)	63 (25.00%)	
	Other (student or retired)	4 (13.33%)	19 (63.33%)	7 (23.33%)	
Job type^*^	Non-medical job	55 (14.25%)	232 (60.10%)	99 (25.65%)	0.023
	Medical job	1 (1.72%)	42 (72.41%)	15 (25.86%)	
Medical insurance	No	31 (13.60%)	134 (58.77%)	63 (27.63%)	0.424
	Yes	25 (11.57%)	140 (64.81%)	51 (23.61%)	
Medical problems^*^	No	47 (16.10%)	166 (56.85%)	79 (27.05%)	0.001
	Yes	3 (2.21%)	103 (75.74%)	30 (22.06%)	
	I don't know	6 (37.50%)	5 (31.25%)	5 (31.25%)	
Knowledge about COVID-19 symptoms^*^	No knowledge	2 (25.00%)	5 (62.50%)	1 (12.50%)	0.007
	Limited knowledge	15 (13.89%)	52 (48.15%)	41 (37.96%)	
	Good knowledge	39 (11.89%)	217 (66.16%)	72 (21.95%)	
Relative developed COVID-19	No	8 (8.99%)	54 (60.67%)	27 (30.34%)	0.346
	Yes	48 (13.52%)	220 (61.97%)	87 (24.51%)	
Lost a relative due to COVID-19	No	50 (13.62%)	219 (59.67%)	98 (26.70%)	0.135
	Yes	6 (7.79%)	55 (71.43%)	16 (20.78%)	

^*^Gender, job type, medical problems, and knowledge about COVID-19 were found to be statistically significant < 0.05 in vaccine confidence.

**Figure 2:**
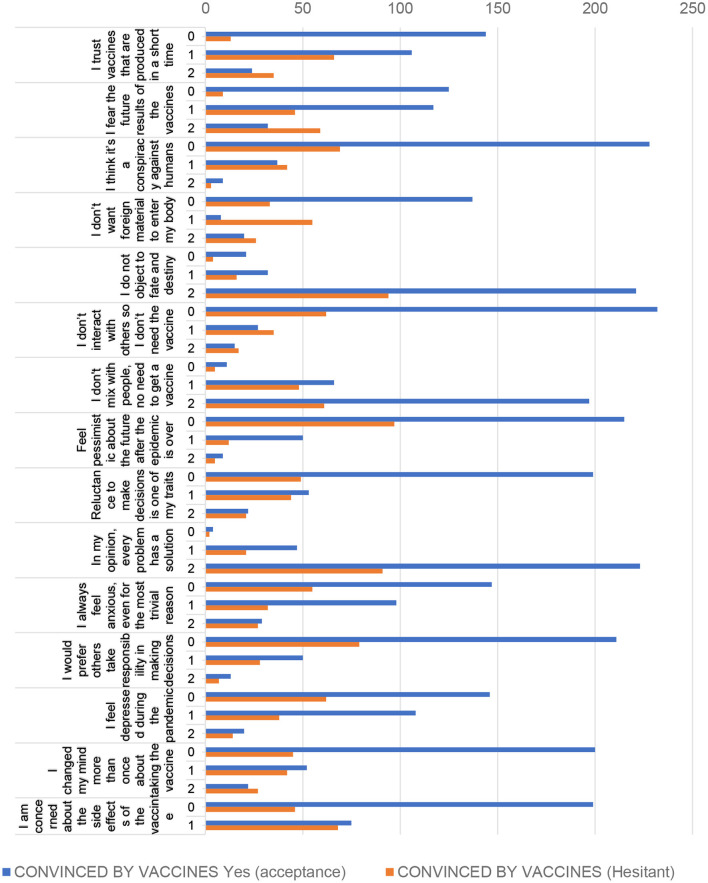
The frequency distribution and chi-squared analysis of the influence of personal characteristics and vaccine confidence. 0 = Do not agree, 1 = Somewhat agree, 2 = Agree.

As shown in Figure 2, the personal characteristics associated with vaccine confidence among participants were as follows: trusting vaccines produced in a short time (*p* = 0.01); being afraid of the future results of the vaccines (*p* = 0.01); do not want a foreign material to enter the body (*p* = 0.01); do not interact with others, do not need the vaccine (*p* = 0.01); do not mix with people a lot, so there is no need for a vaccine (*p* = 0.003); and reluctance to make decisions (*p* = 0.01). Moreover, they always felt anxious, even for the most trivial reasons (*p* = 0.02), felt depressed during the pandemic (*p* = 0.042), changed their mind more than once about the decision to take the vaccine (*p* = 0.01), and were concerned about the side effects of the vaccine that they heard from a friend or relative (*p* = 0.01). All of these factors were significantly associated with vaccine confidence (*p* < 0.05).

Other factors did not have a statistically significant effect on vaccine confidence (*p* > 0.05), including the following: I do not object to fate and destiny (*p* = 0.568); I feel pessimistic about the future even after the epidemic is over (*p* = 0.057); every problem has a solution (*p* = 0.989); and I would prefer that others take responsibility for me in making decisions (*p* = 0.486).

### 3.4. The social and cultural motivations behind individual's vaccine confidence

As shown in Table 4, the social and cultural motivations that influenced vaccine confidence among participants were as follows: free vaccines (*p* = 0.01); media campaigns (*p* = 0.02); reading about vaccines (*p* = 0.01); leaders received vaccines (*p* = 0.01); relatives received vaccines (*p* = 0.03); being obliged to work (*p* = 0.01); religious/God commanded (*p* = 0.026); trusting medical policies (*p* = 0.01); getting back to normal (*p* = 0.01); and getting rid of protective measures (*p* = 0.013). All of these factors were significantly associated with vaccine confidence (*p* < 0.05). Factors that were not significantly associated (*p* > 0.05) with vaccine confidence were: death of a relative due to the virus (*p* = 0.524); fear of social abuse (*p* = 0.489); fear of death from the virus (*p* = 0.111); and traveling/tourism (*p* = 0.111).

**Table 4:** The frequency distribution and chi-squared analysis of social and cultural motivations behind individual's vaccine confidence.

**Variable**		**Convinced about vaccines**			**P-value**
		**No** **(refusal)**	**Yes** **(acceptance)**	**To some** **extent** **(hesitant)**	
Free vaccine	No	20 (21.51%)	9 (9.68%)	64 (68.82%)	0.001
	Yes	115 (36.62%)	159 (50.64%)	40 (12.74%)	
Media campaigns	No	22 (26.19%)	7 (8.33%)	55 (65.48%)	0.002
	Yes	118 (36.53%)	156 (36.53%)	49 (15.17%)	
Death of relative due to virus	No	27 (21.43%)	2 (1.59%)	97 (76.98%)	0.524
	Yes	246 (87.54%)	28 (9.96%)	7 (2.49%)	
Read about vaccine	No	28 (26.67%)	1 (0.95%)	76 (72.38%)	0.001
	Yes	160 (52.98%)	114 (37.75%)	28 (9.27%)	
Leaders received vaccine	No	25 (25.77%)	4 (4.12%)	68 (70.10%)	0.001
	Yes	128 (41.29%)	146 (47.10%)	36 (11.61%)	
Relatives received + vaccine	No	23 (29.11%)	6 (7.59%)	50 (63.29%)	0.003
	Yes	171 (52.13%)	103 (31.40%)	54 (16.46%)	
Fear social abuse	No	27 (21.09%)	2 (1.56%)	99 (77.34%)	0.489
	Yes	251 (89.96%)	23 (8.24%)	5 (1.79%)	
Traveling tourism	No	22 (23.16%)	7 (7.37%)	66 (69.47%)	0.111
	Yes	157 (50.32%)	117 (37.50%)	38 (12.18%)	
Work-mandated vaccine	No	23 (18.70%)	6 (4.88%)	94 (76.42%)	0.001
	Yes	265 (93.31%)	9 (3.17%)	10 (3.52%)	
Religion (God commanded)	No	13 (20.97%)	16 (25.81%)	33 (53.23%)	0.026
	Yes	65 (18.84%)	209 (60.58%)	71 (20.58%)	
Trust in medical policies	No	28 (29.47%)	1 (1.05%)	66 (69.47%)	0.001
	Yes	120 (38.46%)	154 (49.36%)	38 (12.18%)	
Return to normal	No	20 (31.25%)	9 (14.06%)	35 (54.69%)	0.001
	Yes	77 (22.45%)	197 (57.43%)	69 (20.12%)	
Fear of death from the virus	No	28 (22.58%)	1 (0.81%)	95 (76.61%)	0.111
	Yes	235 (83.04%)	39 (13.78%)	9 (3.18%)	
To get rid of protective measures	No	21 (24.14%)	8 (9.20%)	58 (66.67%)	0.013
	Yes	127 (39.69%)	147 (45.94%)	46 (14.38%)	

Hesitancy percentage were as follows based on the following factors: free vaccines (68.82% no vs. 12.74% yes); media campaigns (65.48% no vs. 15.17% yes); death of a relative (no 76.98% vs. 2.49% yes); reading about the vaccine (no 72.38% vs. 9.27% yes); leaders received vaccines (no 70.10% vs. 11.61% yes); relatives received vaccines (no 63.29% vs. 16.46% yes); and fear death due to the virus (no 76.61% vs. 3.18% yes), as seen in Table 4.

## 4. Discussion

Attempts at widespread COVID-19 immunization have been hindered by rejection, hesitancy, rumors, and suspicions. Hesitation about vaccinations may be influenced by beliefs and attitudes about COVID-19, such as the impact of the virus on the person's life, severity of the virus, immunity, thoughts, and attitudes about the vaccine itself, such as vaccine novelty, efficacy, and adverse effects (16). The present study evaluated vaccine hesitancy and sociocultural characteristics to determine what social factors influenced the Saudi population's attitude toward COVID-19 vaccination, given that vaccine acceptance varies by sociodemographic factors (4). The results of the present study revealed that more than half of the respondents (61.7%) were vaccine confident, compared to those who were hesitant (25.7%), and not convinced (12.6%). According to a study conducted in the U.S., the probability of Americans receiving a COVID-19 vaccination was as follows: very likely (52%), somewhat likely (27%), not likely (15%), and definitely not (7%) (15). In another part of the world, Indonesia, vaccine acceptance is quite high (93.3%) (17). Demographic characteristics such as sex, job type, medical problems, and knowledge about COVID-19 symptoms were found to be associated with variability in vaccine acceptance. Social life, education, job permanence, and medical insurance, someone close contracted COVID-19, and lost someone close due to COVID-19 are factors which do not have a statistically significant association to the vaccine confidence. This information may help governments, policymakers, healthcare providers, and international organizations better target COVID-19 immunization campaigns. In the present study, healthcare workers were found to have a higher acceptance of the vaccine than the general public, in contrast to a previous study in which the general public showed better acceptance of the vaccine than healthcare workers in Saudi Arabia (18).

Most respondents who accepted vaccines shared the following characteristics: aged 41–51 years; married; had a job in the medical field; had medical problems; had a diploma-level education; had medical insurance; had good knowledge about COVID-19 symptoms; and lost a close relative due to COVID-19. These factors may be related to people with high personal maturity and may be more realistic in certain populations than in others. Additionally, respondents aged 41–51 years were more willing to be vaccinated than younger adults. The younger adults showed lower acceptance (48.0%) of the vaccine in Saudi Arabia, as reported in a recent study (19, 20). Married individuals with higher education were more willing to be vaccinated, which is similar to a previous study involving the Saudi population (20). Participants who had good knowledge about the virus, such as people in the medical field, also showed a higher acceptance of the vaccine. These categories of people are expected to have better access to accurate information about the side effects and the advantages and disadvantages of COVID-19 vaccination.

The most vaccine hesitant were those aged 21–30 years and widows. Participants without a permanent job, no medical insurance, limited understanding of COVID-19 symptoms, no close family members affected by COVID-19, and those who had not read about the vaccine and did not fear death from the virus were also more hesitant. These factors are associated with a lower level of knowledge about the vaccine, a low level of maturity, and personal circumstances. There was still a high percentage of hesitancy, even when the participants knew free vaccines were available, were exposed to media campaigns, had leaders who were vaccinated, and had relatives who were vaccinated. Previous studies have discovered promising strategies for increasing confidence and decreasing vaccine hesitancy in a variety of contexts (21, 22).

Efficacy and safety were among the primary reasons for vaccine hesitancy in previous studies. Polack et al. (23), for example, concluded that a two-dose regimen of the Pfizer BioNTech COVID-19 vaccine (BNT162b2) was 95% effective in preventing COVID-19 in adults aged 16 and older (23). As such, some concerns regarding the vaccine's long-term efficacy and safety are valid. The ongoing pandemic has significantly impacted people's lives and jobs, affecting their health and welfare. One-third of respondents in Saudi Arabian research reported experiencing moderate-to-severe depression, anxiety, and stress as a result of the pandemic (24). As a result, it is unsurprising that the perceived benefits of vaccination, such as decreased anxiety, decreased risk of COVID-19 infection, and simplification of prophylactic activities, were sufficient to reduce participants' hesitancy and raise their confidence about vaccines.

The results of the present study show the social and cultural motivations that influenced vaccine confidence among participants who were free vaccine, media campaigns, reading about the vaccine, leaders receiving the vaccine, relatives receiving the vaccine, being obliged by work, religious/God commanded, trust in medical policies, getting back to normal, and getting rid of protective measures. All these factors had a statistically significant association with vaccine confidence in the current study. Certain groups, such as healthcare workers and businesses that demand in-person presence or serve vulnerable populations, claim that vaccination is mandatory. Students, professors, schoolchildren, teachers, and staff may require vaccines to return to a safe educational environment (25). In the present study, vaccine confidence increased among participants because of trust in medical policies, obligation to take the vaccine, and leaders receiving the vaccine. However, a global survey revealed that employer-mandated immunization increased vaccination rejection among participants of various nationalities. Therefore, voluntary vaccination promotion is preferable. The participation of reputable non-governmental organizations and community-based organizations is critical for establishing trust in the COVID-19 immunization program (26).

Based on the Hofstede model, used to explain the socio-cultural context behind individuals' vaccine hesitancy (12), the causes of uncertainty were built upon personal characteristics around what is or is not trustworthy. On the individualism side, everyone is expected to look after him/herself as an extremely fundamental cultural dimension in forming decisions toward the vaccine. Additionally, uncertainty avoidance differs from risk avoidance (27) in that it addresses society's tolerance for ambiguity. This indicates the extent to which culture causes members to feel uncomfortable or comfortable in unstructured situations. Uncertainty-averse cultures attempt to reduce the possibility of such situations by enforcing strict behavioral codes, laws, and rules; condemning deviant opinions; and believing in absolute truth. In the Saudi case, political trust, loyalty, quality strategic policies, and religious faith were behind raising confidence in the reliability of the vaccine.

Unexpectedly, however, media language that fosters “vaccine refusal” among the population has not affected their decisions, at a time when the media was already disoriented by overwhelming information on the ongoing pandemic and the affectless of the vaccine (4). While access to health information was demanded, the phenomenon named “infodemic” that includes conflicting, confusing, and unreliable information, including misinformation and disinformation (28) that encourage vaccine hesitancy was widely spread through social media as fertile soil for emotions rather than logic, Saudis tended to be more logical than emotional. Overall, the results of the present study illustrate that Saudi traditions have slightly shifted to a medium-term orientation by being adaptable to changed circumstances, learned from previous lessons and other countries, and decreasing gratification of needs and regulating it by means of strict social norms when it comes to health care. However, this study did not document any adverse events, including psychological issues, which may be one of the reasons for the poor acceptance of the vaccine. Therefore, the psychological issues need to be further investigated.

The primary limitation to the present study was sample recruitment, due to the cross-sectional nature of this investigation. The investigators for this study used a purpose sample and anonymous snowball sampling, to collect data, which assisted in increasing the number of participants and preventing selection bias. Second, differences in COVID-19 vaccination acceptance between urban and rural areas were not evaluated in the present study. This is a critical sociodemographic predictor of vaccine hesitancy, as has been demonstrated in multiple studies.

## 5. Conclusion

The present study provided an assessment of the Saudi population's COVID-19 vaccine hesitancy, and demonstrates that most participants were confident about the vaccine. The results of the present study indicated a high prevalence of vaccination acceptance among the Saudi population, with some sociodemographic and cultural factors that might influence the hesitancy rate. Sociodemographic factors, such as gender, job type, medical problems, and knowledge about COVID-19 symptoms, were found to be associated with higher vaccine acceptance. Free vaccines, media campaigns, reading about the vaccine, leaders receiving the vaccine, relatives receiving the vaccine, work-mandated vaccination, religion, trust in medical policies, returning to normal life, and getting rid of protective measures were found to decrease hesitancy. Policymakers, therefore, should encourage scientific research to demonstrate vaccine safety over the long term. Vaccine literacy initiatives should be tailored to the high-priority population's levels of health, scientific, and general literacy. Vaccination hesitancy reduction strategies should be adopted after carefully concentrating on the cultural factors of the target population, to address community-specific vaccination misconceptions.

## Data availability statement

The original contributions presented in the study are included in the article/supplementary material, further inquiries can be directed to the corresponding author.

## Ethics statement

The studies involving human participants were reviewed and approved by Umm Al-Qura University Ethical Committee, ethical approval number: HAPO-02-K-012-2021-03-595. The patients/participants provided their written informed consent to participate in this study.

## Author contributions

All authors listed have made a substantial, direct, and intellectual contribution to the work and approved it for publication.
